# Elevated prevalence of multidrug-resistant gram-negative organisms in HIV positive men

**DOI:** 10.1186/s12879-017-2286-z

**Published:** 2017-03-13

**Authors:** Claudia Reinheimer, Oliver T. Keppler, Christoph Stephan, Thomas A. Wichelhaus, Imke Friedrichs, Volkhard A. J. Kempf

**Affiliations:** 1 Institute for Medical Microbiology and Infection Control, University Hospital Frankfurt, Paul-Ehrlich-Str. 40, 60596 Frankfurt am Main, Germany; 2 University Center for Infectious Diseases, University Hospital Frankfurt am Main, Frankfurt am Main, Germany; 3 Institute for Medical Virology, University Hospital Frankfurt, Frankfurt am Main, Germany; 4 Present address: Max von Pettenkofer-Institute for Hygiene and Clinical Microbiology, Virology, Ludwig Maximilians-University, Munich, Germany; 5 Department for Internal Medicine II/Infectious Diseases, University Hospital Frankfurt, Frankfurt am Main, Germany

**Keywords:** Emerging pathogens, Multidrug-resistant gram-negative bacteria, Infection control, Epidemiology

## Abstract

**Background:**

Routes of transmission of multidrug-resistant gram-negative organisms (MDRGN) are not completely understood. Since sexual transmission of MDRGN might represent a potential mode that has not been noticed so far, this study evaluated transmission of MDRGN in HIV positive men.

**Methods:**

Between November 2014 and March 2016, we retrospectively investigated the MDRGN prevalence in rectal swabs of *n* = 109 males tested positive for HIV (HP). These findings were compared to the MDRGN prevalence in *n* = 109 rectal swabs in age-matched males tested negative for HIV (HN) within the same period. According to the infection control protocol of University Hospital Frankfurt, Germany (UHF), patients admitted to intensive/intermediate care units have to be screened for MDRGN on day of admittance. Patients without HIV testing or MDRGN screening were excluded.

**Results:**

MDRGN prevalence in rectal swabs was significantly higher (*p* = 0.002) in male HP (23.9%; 95% confidence interval 16.2–32.9%) than in age-matched male HN (8.3%; 3.8–15.1%). In total, 35 MDRGN species were detected. The most frequent MDRGN species was *Escherichia coli* with resistance due to ESBL expression and additional resistance to fluoroquinolones with *n* = 25/35 (71.4%; 53.7–85.4%). Thereof, *n* = 19/26 (73.1%; 52.2–88.4%) were detected in HP and *n* = 6/9 (66.7%; 29.9–92.5%) in HN, respectively.

**Conclusions:**

Prevalence of MDRGN is significantly higher in male HIV positive than in male HIV negative individuals. This might indicate sexual transmission of MDRGN within the male HIV positive population. As treatment options in case of MRGN infections are limited, prevention of MDRGN transmission is strongly emphasized.

## Background

Multidrug–resistant gram–negative organisms (MDRGN), which have previously been defined as *Enterobacteriaceae* with extended spectrum beta–lactamase (ESBL)–phenotype, and *Enterobacteriaceae, Acinetobacter baumannii* and *Pseudomonas aeruginosa* resistant against piperacillin, any 3rd/4th generation cephalosporin, and fluoroquinolones +/– carbapenems [1], are frequent causes of community and healthcare-associated infections [2, 3]. In recent years, carbapenems became the drugs of choice for the treatment of invasive MDRGN infections [4]. At the same time, the consumption of carbapenems has been increased dramatically [5] and the worldwide prevalence of carbapenemase-producing organisms soon after was found to be elevated [6, 7]. While the explosive spread of carbapenemases has previoulsy been attributed to several factors, such as increasing administration of carbapenems or contact to the health care system in high-prevalence countries (HPC) [8–10], global spread might also be favored by plasmid encoded resistance genes transfer [11]. However, comprehensive knowledge of the ways of transmission is still lacking. Future concise considerations on the spread of MDRGN should therefore also evaluate routes of transmission that have not been noticed so far. We hypothesize that microbiota, with MDRGN might be part of it, is exchanged during mucous membranes contacts, *e.g.* sexual intercourse, Although the primary route of MDRGN transmission might be different, sexual transmission should therefore not dismissed. This hypothesis has previously been confirmed by investigations on the sexual transmission of *Human Herpes Virus Type 8*, *E. coli* O117:H7 and *Shigella spp.*, although the primary route of these agents’ transmission is via saliva or feco-orally, respectively [12–14]. It additionally should be taken into account that it has previously been reported that the risk to acquire “classical” sexually transmitted diseases (STD) such as gonorrhea or syphilis, or infection with the *Human Immunodeficiency Virus* (HIV) is higher in cohorts of men who have sex with men (MSM) than for the general population [15, 16]. This has also been demonstrated for Germany [17, 18]. With regard to these aspects, pre– or co–existing STD (*e.g.* HIV infection) have been demonstrated to be suitable parameters to identify individuals with previous or current unprotected, “risky” sexual intercourse. We therefore hypothesize that MDRGN prevalence might be higher in patient groups with HIV infection compared to patient groups tested negative for HIV. In light of the persistent global spread of MDRGN, possibly with a number of different routes, the role of sexual transmission has to be clarified. We present the first study addressing this possible transmissibility of MDRGN by sexual intercourse.

## Methods

### MDRGN screening policy at University Hospital Frankfurt, Germany

According to German infection protection law it is mandatory for hospitals to execute documented infection control strategies to prevent the transmission of infective agents and protect patient health. At University Hospital Frankfurt, Germany (UHF), several defined patient risk groups are screened for MDRGN and methicillin resistant *Staphylococcus aureus* (MRSA) on day of admittance, such as patients admitted to intensive/intermediate care units (ICU/IMC) at UHF. This means, that each patient will get screened in case of admittance to ICU/IMC at UHF independently from his medical diagnosis.

### Patients and specimens

We retrospectively identified and included adult individuals having had both a rectal screening for MDRGN *and* HIV testing between November 2014 and March 2016. Patients having had only one of the parameters (screening for MDRGN *or* testing for HIV) were excluded from this study. Patients tested positive for HIV and having had a rectal screening for MDRGN qualified for the case cohort, hereinafter referred to as HP. *Vice versa*, patients tested negative for HIV and having had a rectal screening for MDRGN qualified for the control cohort, hereinafter referred to as HN.

Identification of cases (HP) and controls (HN) was performed as follows. First, individuals serologically tested positive for HIV and admitted to the department for Internal Medicine II/Infectious Diseases at UHF were identified. Subsequently, these patients were evaluated for being routinely screened for MDRGN during the observation period according to the infection control protocol of UHF. Control patients who were tested serologically negative for HIV and admitted to any department at UHF were also evaluated for being routinely screened for MDRGN during the observation period. Cases and controls were matched on +/- 2 years. By this approach, *n* = 109 male HP and male HN each qualified for this study. Additionally, digital patient data files were evaluated to stratify patients’ general groups of diseases. Furthermore, the stadium of HIV infection was defined according to CDC criteria [19] of all the patients admitted to the department of Internal Medicine II/Infectious Diseases. With regard to the pilot character of this study and small number of individuals who meet inclusion criteria, co-morbidities and previous antibiotic use were not considered in sampling.

### Detection of MDRGN and molecular resistance analysis

The results for the patients’ MDRGN status were retrospectively investigated. For the investigation, we performed a proven methodical procedure that has formerly been published [1]. During the retrospective observation period, all laboratory testing procedures were performed by the Institute for Medical Microbiology and Infection Control at UHF under strict quality–controlled criteria (laboratory accreditation according to ISO 15189:2007 standards; certificate number D–ML–13102–01–00, valid through January 25th, 2021). Rectal swabs were collected using culture swabs with Amies collection and transport medium (Hain Lifescience, Nehren, Germany) and streaked onto selective CHROMagar™ ESBL plates (Mast Diagnostica, Paris, France). Identification of presumed MDRGN species was done by matrix-assisted–laser desorption ionization–time of flight analysis (MALDI–TOF; VITEK MS, bioMérieux, Nürtingen, Germany). Antibiotic susceptibility testing was performed according to Clinical and Laboratory Standards Institute (CLSI) guidelines using VITEK 2 and/or antibiotic gradient tests (bioMérieux) where necessary. In case of gram-negative carbapenem-resistant isolates, detection of genes encoding carbapenemases were routinely performed via PCR analysis and subsequent sequencing from carbapenem resistant *Enterobacteriaceae* including the *bla* genes for the following carbapenemases: NDM, VIM, IMP, OXA–48, and KPC [10, 11, 20].

### Detection of HIV-1/2 antibodies

Considering that HIV positivity has been suggested to be a marker for a risky sexual behaviour [20], we retrospectively evaluated the HIV status of each individual by examination of the patient files. HIV testing was only performed if medically indicated by physicians at UHF. Laboratory HIV testing was performed by Institute for Medical Virology at UHF under strict quality–controlled criteria (laboratory accreditation according to ISO–15189:2007 standards; certificate number D–ML–13102–03–00 valid through 08.12.2018). All serum samples had been previously screened with the HIV antigen/antibody (Ag/Ab) Combo Screening Assay (Abbott, Delkenheim, Germany; Architect System; REF 4 J27) working with the Architect system detecting HIV–1/2 antibodies and HIV–1 p24 antigen. Samples with reactive screening assay results (positive, indeterminate) were further evaluated using the western blot New Bio-Rad LAV–Blot I and LAV–Blot II (Bio–Rad Laboratories GmbH, München, Germany), which uses native HIV proteins. The assay was performed as outlined in guidelines provided by the manufacturer.

### Statistical analysis

Sample size of *n* = 109 individuals per group proved to be adequate by a power = 0.95 and Cohen’s *d* = 0.5. Chi squared test was performed for statistical analysis. 95% confidence intervals (95% CI) for frequencies were calculated based on binomial distribution and used to confirm statistical significance. *P*-values ≤ 0.05 were considered as statistically significant.

## Results

In the current study, we evaluated *n* = 109 male HP and *n* = 109 male HN, respectively, admitted to UHF between November 2014 and March 2016. Both the male HPs’ and male HNs’ median age was 46 years, the standard deviation being 10.8 and 11.0 years, respectively.

**Fig. 1:**
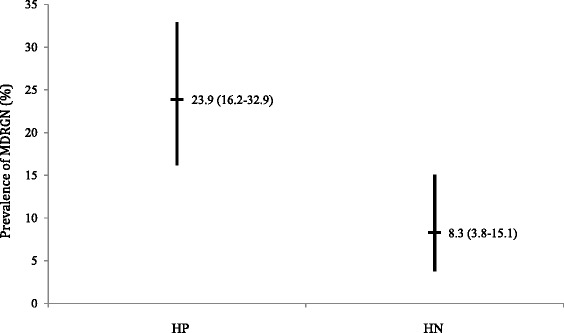
Prevalence (%) of multidrug-resistant gram-negative organisms (MDRGN) in patients tested positive (HP) and negative for HIV (HN). 95% confidence intervals are marked as ranges; percentage and 95% confidence intervals are given

Table 1 shows the general groups of diseases HP and HN they were admitted for to UHF. In HP, the most frequent group were patients admitted to the department of Internal Medicine II/Infectious Diseases with 82.6% (*n* = 90/109). HN were most frequently admitted to UHF due to oncological diseases with 39.4% (*n* = 43/109). Among male HP and male HN, a total of *n* = 35 MDRGN isolates were detected. Thereof, *n* = 26/109 and *n* = 9/109 rectal swabs were positive for any MDRGN in male HP and male HN, respectively, which resulted in an overall MDRGN prevalence of 23.9% (16.2–32.9) in male HP and a significantly (*p* = 0.002) lower MDRGN prevalence in male HN with 8.3% (3.8–15.1). Looking into the single proportions of the detected MDRGN, no significant differences were detected between male HP and HN patients, respectively (Fig. 1).

**Table 1:** General group of diseases HIV positive (HP) patients and HIV negative (HN) patients were admitted for to University Hospital Frankfurt, Germany (UHF), as given by the digital patient data files

			HP	HN
General characteristics				
Number of patients (n)			109	109
Male (%)			100	100
Mean age and standard deviation (years)			46 (10.8)	46 (11.0)
General group of diseases patients were admitted for to UHF				
Department for	Infectious diseases (n;%)		90 (82.6)	12 (11.0)
	Stadium of HIV infection ^a^(n;%)			
		A1	8; 8.9	-
		A2	16; 17.8	
		A3	5; 5.6	
		B1	-	
		B2	4; 4.4	
		B3	7; 7.8	
		C1	-	
		C2	5; 5.6	
		C3	40; 44.4	
		unknown	5; 5.6	
	Hematology/Oncology (n;%)		6; 5.6	43 (39.4)
	Gastroenterology (n;%)		3; 2.8	14 (12.8)
	Pneumology (n;%)		1; 0.9	2 (1.8)
	Cardiology (n;%)		-	3 (2.7)
	Nephrology (n;%)		-	7 (6.4)
	Rheumatology (n;%)		-	1 (1)
	Neurology		5; 4.6	5 (4.6)
	General surgery and traumatology (n;%)		4; 3.7	21 (19.3)
	Neurosurgery (n;%)		-	1 (1)

*Abbreviations*: *UHF* University Hospital Frankfurt am Main, Germany; *HIV* human immunodeficiency virus

^a^as defined by CDC [19]: A = asymptomatic, acute HIV, or persistent generalized lymphadenopathy; B = symptomatic conditions, not A or C; C = AIDS-indicator conditions; “1” CD4^+^ cell count ≥ 500/μl; “2” = CD4^+^ cell count 200-499/μl; “3” = CD4^+^ cell count <200/μl (in detail see [19])

The most frequently detected species within the MDRGN isolates (*n* = 35 in total) was *E. coli* with resistance due to ESBL expression and additional resistance to fluoroquinolones (*E. coli* ESBL/FQ) with *n* = 25/35 (71.4%; 53.7–85.3). Thereof, *E. coli* ESBL/FQ was detected in *n* = 19/26 (73.1%; 52.2–88.4%) of male HP and in *n* = 6/9 (66.7%; 29.9–92.5%) of male HN (*p* = 0.713), respectively. All MDRGN detected in male HP and male HN are depicted by in Table 2. None of the bacterial species *P. aeruginosa* or *A. baumannii* and neither, no resistance to carbapenems were detected in any of the isolates from male HN nor any of HP.

**Table 2:** Prevalence of multidrug-resistant gram-negative organisms in males tested positive for HIV and males tested negative for HIV

	HP	HN
Number of patients (n)	109	109
Median age and standard deviation (years)	46; 10.8	46; 11.0
Tested positive for MDRGN (%; 95% CI; n)	23.9; 16.2–32.9; 26	8.3; 3.8–15.1; 9
Total number of MDRGN isolates (n)	35	
Thereof (n)	26	9
*E. coli* ESBL/FQ (%; 95%CI; n)	73.1; 52.2–88.4; 19	66.7; 29.9–92.5; 6
*E. coli* ESBL (%; 95%CI; n)	23.1; 8.9–43.6; 6	11.1; 0.3–48.3; 1
*K. pneumoniae* ESBL/FQ (%; 95%CI; n)	3.8; 0.0–19.6; 1	22.2; 2.8–60.0; 2
MDRGN with resistance to carbapenems	0	0

*Abbreviations*: *HIV* human immunodeficiency virus; *HP* males tested positive for infection with HIV; *HN* males tested negative for infection with HIV; *MDRGN* multidrug-resistant gram-negative organism. *E. coli* ESBL: resistant due to ESBL expression; *E. coli*/*K. pneumoniae* ESBL/FQ: ESBL and resistance to fluoroquinolones

## Discussion

In light of the explosive global spread of MDRGN, attention must be directed to potential routes of transmission that have not been recognized so far. Several routes of transmission, e.g. traveling in HPC [8], antibiotic selective pressure [5], or transmission by livestock [21] have previously been demonstrated. However, to our knowledge this is the first study focusing on the potential transmission of MDRGN in male HIV positive individuals.

The prevalence of MDRGN in male HN amounted to 8.3% (3.8–15.1; Table 2). This value does not substantially deviate from the prevalence of ESBL of about 6.3% in clinical specimen, which has formerly been described to be 6.3% by a German cross–sectional study [22]. However, the prevalence of MDRGN in rectal swabs of male HP (23.9%; 16.2–32.9) exceeded the value investigated by *Valenza* et al. [22] by almost three-fold. These findings suggest that HIV positive men might be more susceptible to acquire MDRGN. Furthermore, our findings indicate a pre–dominance of *E. coli* ESBL/FQ (Table 1), it might therefore be interesting to characterize these *E. coli*-isolates isolated from male HP further for expression of pathogenicity factors, *e.g.* type 1 fimbriae (f*imH*), pili associated with pyelonephritis (*pap*), S and F1C fimbriae (*sfa* and *foc*), afimbrial adhesins (*afa*), cytotoxic necrotizing factor (*cnf*), hemolysin (*hly*) or aerobactin (*aer*) using molecular techniques [23] and subsequently compare these findings with isolates collected from HN. We therefore feel it is warranted to further characterize MDRGN isolates isolated from HP and HN by molecularbiological methods, preferentially by *whole genome sequencing* (WGS).

Although no significant differences in proportion of any single MDRGN species were observed (Table 2), this might indicate that the sexual route of transmission is apparently not preferred by any particular MDRGN species. It rather might be interpreted as a general inherent risk to acquire a MDRGN. Since the sexual route of transmission of MDRGN has not been suggested so far, this is as important finding of this study. However, several epidemiological events give an impression that sexual spread of organisms is possible, although their preferred way of transmission is reported to be non-sexual: *Shigella sonnei*, which is either none of the “classical” STD-causing organisms, has been shown to spread among men who have sex with men (MSM) in Montréal, Canada, and Germany [24, 25] the verocytotoxin-producing *EHEC* O117:H7 has also been documented to spread among MSM in Great Britain [16] and, as well, no “classical” STD, meningococcal serogroup C diseases has newly been shown to spread among MSM [26, 27]. This outlines that sexual transmission of *Enterobacteriaceae* is distinctly possible via translocation of intestinal bacteria. Sexual transmission of MDRGN is therefore suggested not to be dismissed in future comprehensive considerations on MDRGN transmission prevention.

However, the link between HIV positivity and risky sexual behavior must be interpreted diligently: if positive HIV serology might indicate that the patient has previously had a risky behavior (and HIV was not contracted *e.g.* by blood transfusion), this may have lasted for years up to now, or it may have ended in the meanwhile. The time of carriage of MDRGN might be short [28] and we therefore cannot ascertain that the timeframes between the risky behavior and MDRGN acquisition could coincide. However, at this time we did not see any alternative assessment available to clarify the potential route of sexual transmission.

Concerning that data regarding the history of previous hospital stays nor antibiotic pre–treatment for male HP and male HN were not evaluated, it is not possible to confirm that male HP in the UHF–setting might have had a stronger history of antibiotic pre–treatment than male HN. Considering that all patients enrolled in this investigation matched the requirement to be screened for MDRGN (which is usually limited to patients admitted to ICU or IMC at UHF), this might indicate that both male HP and male HN had a recent history of antibiotic treatment. However, HIV patients in particular might have undergone a prexisiting antibiotic pressure due to their HIV infection (*e.g.* administration of cotrimoxazole to treat *Pneumocystis jirovecii* pneumonia) and might therefore have a higher risk for admittance to hospitals and health care facilities where nosocomial MDRGN transmissions can occur [29]. These aspects might therefore have introduced a source of biases in the selection of patients.

However, not also antibiotic treatment, but also pre–existing co-morbidities also might be an aspect that has to be evaluated in a future setting to round off investigations on conditions of sexual transmission of MDRGN. Since gastrointestinal diseases or co-infections might compromise the gastrointestinal mucosa, this might promote the transmission of MDRGN. As such interrelation has formerly been shown for amebiasis and HIV [30], this aspect should not be neglected.

Furthermore, it remains open to question whether these findings are also valid for women. As urinary tract infections (UTI) are one of the most common (usually endogenous) bacterial infections often triggered by sexual intercourse, and many women suffer from at least one UTI during their lifetime [31], in a future setting the prevalence of MDRGN should be investigated in urogenital and/or vaginal swabs in female cohorts.

## Conclusions

Our study demonstrated that prevalence of MDRGN in HIV positive male individuals is significantly higher than in HIV negative male individuals. Although no significant differences in proportion of any single MDRGN species were observed, this indicates that sexual transmission of MDRGN within the male HIV positive population is possible. With regard to the results of this study, practicing safer sex should be propagated actively further on to reduce not only the spread of many STD-associated pathogens but most likely also MDRGN.

## Abbreviations

CI: Confidence interval
CLSI: Clinical and Laboratory Standards Institute
EHEC: Enterohaemorrhagic *Escherichia coli*
ESBL: Extended spectrum beta–lactamase
ESBL/FQ: ESBL expression and additional resistance to fluoroquinolones
HIV: Human immunodeficiency virus
HN: Males serologically tested negative for HIV
HP: Males serologically tested positive for HIV
HPC: High-prevalence countries
ICU: Intensive care unit
IMC: Intermediate care unit
IMP: Imipenem-hydrolyzing beta-lactamase
KPC: *Klebsiella pneumoniae* carbapenemase
MALDI-TOF: Mass-assisted–laser desorption ionization–time of flight analysis
MDRGN: Multidrug-resistant gram-negative organisms
MSM: Men who have sex with men
NDM: New Delhi metallo-beta-lactamase
PCR: Polymerase chain reaction
STD: Sexually transmitted diseases
UHF: University Hospital Frankfurt, Germany
UTI: Urinary tract infection
VIM: Verona integron-encoded metallo-beta-lactamase
WGS: Whole genome sequencing
